# Statistical quality in RCTs of technology-based rehabilitation after spinal cord injury: A CHAMP-Based Meta-Research Study

**DOI:** 10.1038/s41393-026-01276-0

**Published:** 2026-09-18

**Authors:** Alex Lando, Martina Andò, Lorenza Maistrello, Luisa Cacciante, Giorgia Pregnolato, Sara Federico, Paolo Pillastrini, Marco Tramontano, Andrea Turolla

**Affiliations:** 1 Translational Rehabilitation Sciences Group, Department of Biomedical and Neuromotor Sciences (DIBINEM), Alma Mater Studiorum University of Bologna, Bologna, Italy; 2 IRCCS San Camillo Hospital, Venice, Italy; 3 Healthcare Innovation Technology Lab, IRCCS San Camillo Hospital, Venice, Italy; 4 Insight Research Ireland Centre for Data Analytics, University College Dublin, Dublin, Ireland; 5 School of Electrical and Electronic Engineering, University College Dublin, Dublin, Ireland; 6 Unit of Occupational Medicine, IRCCS University Hospital of Bologna, Bologna, Italy; 7 Professional Development and Implementation of Research in Health Professions Unit, IRCCS Azienda Ospedaliero-Universitaria di Bologna, Bologna, Italy

**Keywords:** Randomized controlled trials, Outcomes research

## Abstract

**Study design:**

Cross-sectional meta-research study.

**Objectives:**

Technology-based rehabilitation (TBR) is increasingly used in spinal cord injury (SCI), with a growing number of randomised controlled trials (RCTs) evaluating its effectiveness. However, inadequate statistical reporting may limit the interpretation, reproducibility, and clinical applicability of findings. This study aimed to evaluate the statistical quality of RCTs investigating TBR interventions in individuals with SCI.

**Methods:**

MEDLINE, CINAHL Complete, Cochrane CENTRAL, PEDro, and Web of Science were searched from 1996 to October 2025. Eligible studies were superiority parallel-arm RCTs involving adults with traumatic or non-traumatic SCI evaluating TBR interventions and explicitly reporting adherence to the Consolidated Standards of Reporting Trials (CONSORT). Statistical quality was assessed using the Checklist for Statistical Assessment of Medical Papers (CHAMP), while methodological quality was evaluated using the Risk of Bias (RoB) 2 tool.

**Results:**

Eight RCTs were included. Overall, CHAMP compliance was 57.9%, with over 40% of items rated as unclear or not fulfilled. Compliance was highest in the design domain (64.6%) and lower in the analysis (58.8%), interpretation (58.9%), and reporting (50.0%) domains. CHAMP scores ranged from 0.30–0.83. According to RoB 2, one study (12.5%) was judged at low risk of bias, five studies (62.5%) raised some concerns, and two studies (25.0%) were judged at high risk of bias.

**Conclusions:**

Statistical quality in RCTs of TBR in SCI is frequently suboptimal despite reported adherence to CONSORT guidelines. Greater transparency in statistical reporting is needed to improve the interpretation, reproducibility, and clinical applicability of rehabilitation research.

**Figure:**
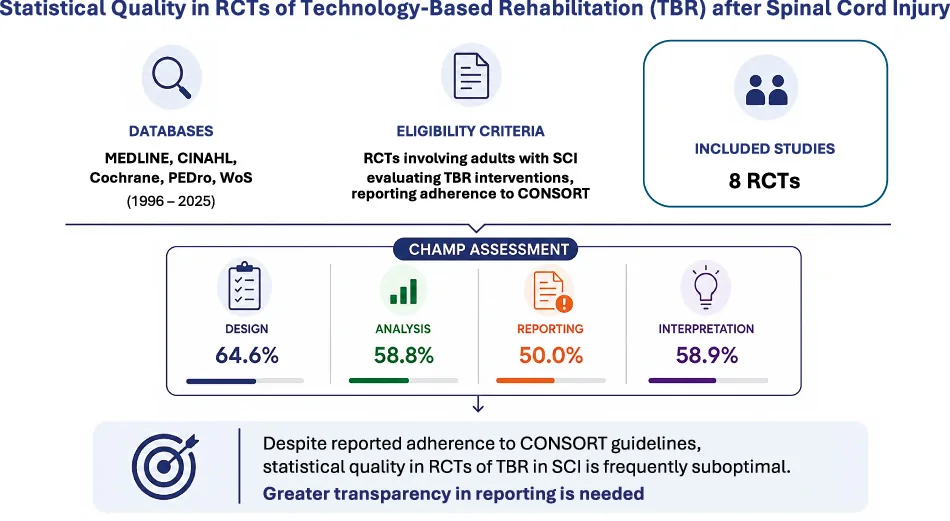

## Introduction

Spinal cord injury (SCI) is a complex neurological condition characterised by partial or complete disruption of motor and sensory pathways, often resulting in long-term impairments in motor control, functional independence, participation, and quality of life [1].

Improving motor function is a primary goal of rehabilitation [2], and technological innovations have been increasingly integrated into clinical practice to support this aim [3].

The rapid evolution and heterogeneity of these approaches have hindered the establishment of a universally accepted definition [4]. Technology-based rehabilitation (TBR) generally encompasses a broad range of interventions, including robotic devices, virtual and augmented reality (VR/AR) systems, functional electrical stimulation (FES), and sensor-based or digital platforms, designed to enhance motor relearning through increased training intensity, task specificity, feedback, and patient engagement [5].

The growing adoption of technology in rehabilitation practice has been paralleled by an increase in randomised controlled trials (RCTs) evaluating TBR in individuals with SCI [6]. Although RCTs represent the gold standard for assessing intervention effectiveness, their value depends critically on the methodological quality, as well as on the transparency of statistical methods and results [7]. Indeed, inadequate statistical reporting may compromise the interpretation of findings, limit reproducibility, and weaken the evidence for clinical decision-making [8].

The Consolidated Standards of Reporting Trials (CONSORT) statement [9] was developed to improve the clarity and completeness of RCT reporting and is widely endorsed by scientific journals. However, although adherence to CONSORT may improve the completeness and transparency of overall trial reporting, it is not specifically designed to evaluate the quality, appropriateness, and transparency of statistical methods and results [10]. Likewise, methodological quality assessment tools such as Risk of Bias (RoB) 2 primarily evaluate threats to internal validity rather than the appropriateness and transparency of statistical methods [11]. In this context, dedicated tools such as the Checklist for Statistical Assessment of Medical Papers (CHAMP) [12] provide a structured framework to evaluate statistical design, analysis, reporting and interpretation of findings. CHAMP specifically addresses key aspects of statistical practice, including hypothesis specification, choice of statistical methods, handling of missing data, reporting of effect sizes and precision, and transparency of analytical decisions [13]. Therefore, CHAMP complements methodological quality assessment by specifically focusing on statistical reporting practices that may not be captured by traditional risk of bias tools.

Given the methodological complexity and heterogeneity of TBR interventions, rigorous and transparent statistical methods and results are essential [14]. However, the extent to which these standards are met in RCTs involving adults with SCI remains unclear [15]. Therefore, this meta-research study aimed to evaluate the statistical quality of these studies that explicitly declare adherence to the CONSORT statement. Using the CHAMP framework, we also aimed to identify common strengths and limitations across statistical design, analysis, reporting, interpretation, and to explore their relationship with methodological quality.

## Methods

A cross-sectional meta-research study design was adopted, with the published trial report as the unit of analysis. The study was reported following the Preferred Reporting Items for Systematic Reviews and Meta-Analyses (PRISMA) principles adapted for meta-research studies [16]. The study protocol was prospectively registered in PROSPERO (CRD420251161874).

### Data sources and searches

A systematic literature search was conducted on October 13, 2025, in the following electronic databases: MEDLINE, CINAHL Complete, Cochrane Central Register of Controlled Trials (CENTRAL), PEDro, and Web of Science. Searches covered the period from 1996, corresponding to the publication of the first CONSORT statement [17], to the search date. Search strategies combined controlled vocabulary terms (e.g., MeSH) and free-text keywords related to SCI, TBR, and RCTs. The full search strategies for all databases are reported in Supplement S1.

### Study selection

Records retrieved through the search were independently screened, based on titles and abstracts, by two reviewers (AL and MA). Full texts of potentially eligible studies were subsequently assessed against the predefined eligibility criteria. Disagreements were resolved by a third reviewer (LC).

We included superiority, parallel-arm RCTs involving adults (i.e., ≥18 years) with traumatic or non-traumatic SCI, investigating TBR interventions aimed at improving one or more of the following outcomes: motor function, mobility, functional independence, or health-related quality of life.

Given the absence of a universally accepted definition, an operational definition of TBR was adopted for study eligibility, based on a recent classification framework [4]. TBR was defined as any intervention in which technological systems constituted a core component of the therapeutic process, actively supporting, guiding, or modulating motor training, rather than being used solely for outcome assessment, monitoring, or passive physical modalities. Accordingly, eligible interventions included robotic or electromechanical devices for upper or lower limb rehabilitation, VR or AR systems, exergaming platforms, non-invasive brain stimulation (NIBS), FES, brain-computer interfaces (BCIs), telerehabilitation platforms, and interactive sensor-based rehabilitation systems.

A mandatory inclusion criterion was the explicit declaration of adherence to the CONSORT statement, indicated by a direct statement in the manuscript, citation, or provision of a CONSORT checklist. The isolated inclusion of a CONSORT flow diagram was not considered sufficient.

Studies were excluded when technology was used exclusively for assessment or monitoring purposes, when interventions consisted solely of passive physical modalities (e.g., Transfer Energy Capacitive and Resistive - TECAR therapy, ultrasound therapy, vibration therapy, shockwave therapy, or magnetotherapy), or when they were based on conventional treadmill training or cycle ergometry without an active, technology-driven control, feedback, or adaptive component. Trials adopting designs other than superiority parallel-arm RCTs (e.g., non-inferiority or equivalence trials, quasi-randomised studies, pragmatic trials, factorial designs, cluster-randomised or crossover trials, multi-arm trials without a clear parallel comparison, pilot or feasibility studies, secondary analyses, protocols, conference abstracts, and non-human studies) were also excluded to ensure methodological homogeneity and comparability in the assessment of statistical quality across studies.

### Data extraction and quality assessment

Data extraction was performed independently by two reviewers (AL and MA) and verified by two other reviewers (LM and GP). Extracted information included bibliographic details, participant characteristics, intervention and comparator descriptions, primary and secondary outcomes, and all data required to conduct the CHAMP assessment and the RoB 2 evaluation.

The primary outcome was the statistical quality, assessed using the CHAMP checklist, which comprises 30 items across four domains: design (items 1–6), analysis (items 7–16), reporting (items 17–23), and interpretation (items 24–30), thereby covering key aspects of statistical practice [13]. As CHAMP is a checklist rather than a quantitative scale, overall statistical quality was operationalised for the purpose of this study as the proportion of fulfilled items out of all applicable CHAMP items, with scores ranging from 0 (no fulfilled items) to 1 (all items fulfilled). This approach was developed ad hoc to enable a quantitative summary of statistical reporting across studies.

Methodological quality was assessed using the RoB 2 tool, applied to the primary outcome as defined by each individual study. The tool evaluates five domains: (1) the randomisation process, (2) deviations from intended interventions, (3) missing outcome data, (4) measurement of the outcome, and (5) selection of the reported result. Within each domain, a series of signalling questions aims to elicit information about features of the trial that are relevant to risk of bias. A proposed judgement about the risk of bias arising from each domain is generated by an algorithm, based on answers to the signalling questions. Responses to these questions are used to derive domain-level judgements, which in turn inform an overall risk-of-bias judgement for the specific study outcome being assessed. Judgement can be ‘Low’ or ‘High’ risk of bias, or can express ‘Some concerns’ [11].

### Data synthesis and analysis

Data synthesis was primarily descriptive. Item-level CHAMP performance was summarised using frequencies and percentages. A continuous CHAMP proportion score (number of fulfilled items divided by the total number of items) was calculated for each study to represent overall statistical reporting quality. For this purpose, items rated as “Yes” were assigned a value of 1, whereas items rated as “No” or “Unclear” were assigned a value of 0, reflecting insufficient reporting. Inter-rater agreement for the CHAMP assessment was evaluated using percentage agreement and the unweighted Cohen’s κ coefficient, considering the three possible ratings for each CHAMP item (Yes, No, and Unclear). Agreement was calculated across all CHAMP item ratings and separately for each CHAMP domain (design, analysis, reporting, and interpretation). Cohen’s κ values were interpreted according to the criteria proposed by Landis and Koch [18]. Exploratory descriptive comparisons were conducted to examine how CHAMP scores were distributed across studies with different overall RoB 2 judgements. No formal statistical tests of association were performed due to the small number of included studies.

## Results

### Study selection

**Fig. 1:**
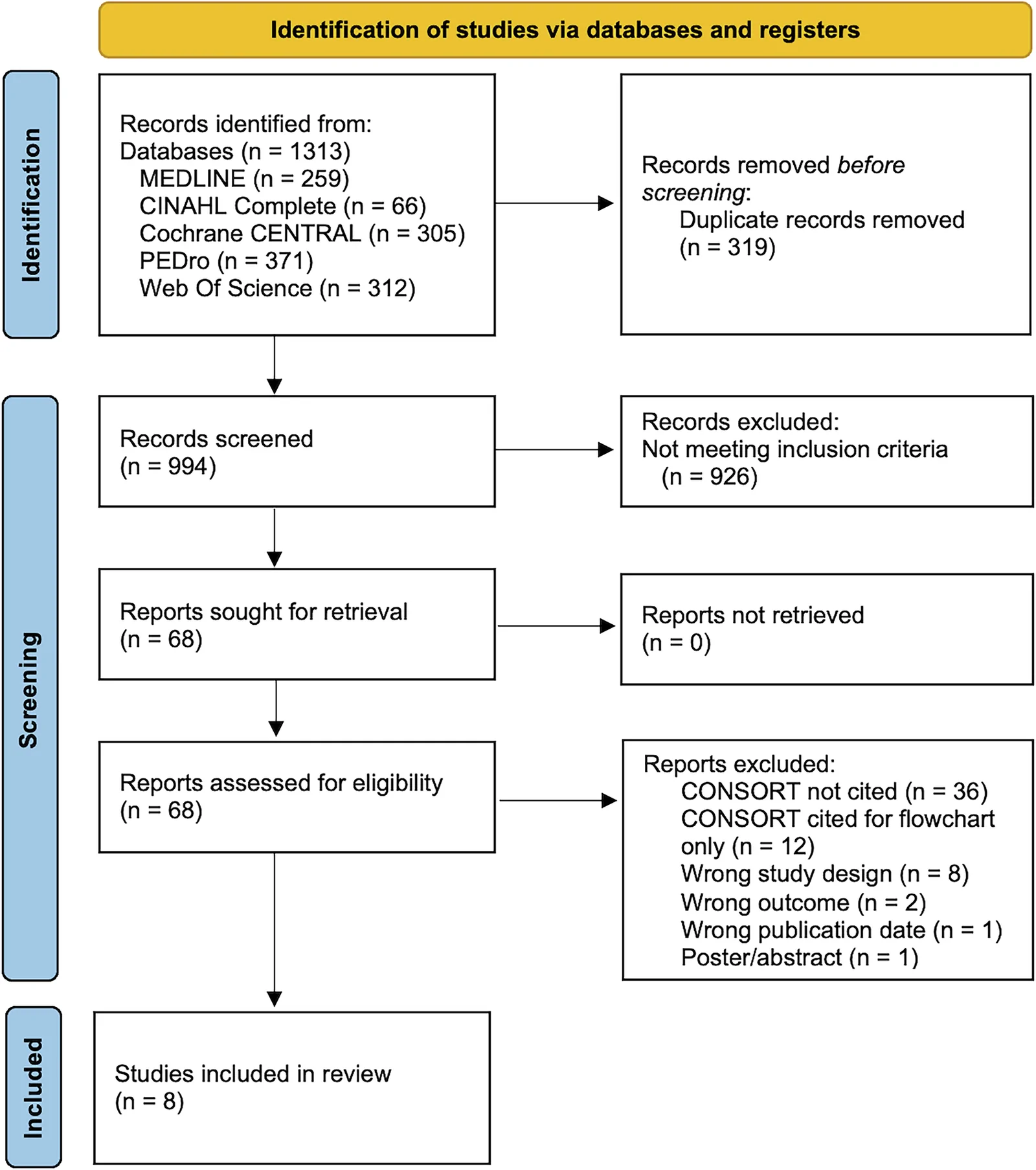
PRISMA flow diagram of the study selection process.

The database search yielded a total of 1313 records. After removal of 319 duplicates, 994 records were screened at the title and abstract level, of which 926 were excluded for not meeting the inclusion criteria. The remaining 68 full-text articles were retrieved and assessed for eligibility. Following full-text evaluation, eight studies met the predefined inclusion criteria and were included in the meta-research analysis. The study selection process is illustrated in the PRISMA flow diagram (Fig. 1).

### Characteristics of included studies

The characteristics of the included studies are summarised in Table 1. The eight included RCTs were published between 2013 [19] and 2025 [20] and involved a total of 394 participants with SCI, with sample sizes ranging from 23 [21] to 161 [22] participants per study. Across trials, 193 participants were allocated to TBR interventions and 201 to control conditions. Six studies [19, 22–26] enrolled individuals with chronic SCI, while two studies [20, 21] included participants in the subacute phase. Neurological levels ranged from cervical to lumbar, and seven trials [19–23, 25, 26] focused exclusively on individuals with motor-incomplete SCI (American Spinal Injury Association Impairment Scale, AIS C-D), whereas one trial [24] also included participants with complete injuries.

**Table 1:** Characteristics of the eight RCTs included in the meta-research analysis. An asterisk (*) indicates outcomes explicitly reported as primary by the original study.

Author (year)	Publication journal	CONSORT	Sample size (TBR/ Control)	SCI characteristics	TBR intervention	Comparator	Outcome measures
Comino-Suárez (2025) [20]	Journal of NeuroEngineering and Rehabilitation	2010	27 (14/13)	Subacute; C2–T11; AIS C–D	tSCS + robotic-assisted gait training (Lokomat®)	Sham tSCS + robotic-assisted gait training (Lokomat®)	LEMS*; Dynamometry; 10MWT; TUG; 6MWT; SCIM-III; WISCI-II; MEPs
Gil-Agudo (2023) [21]	Journal of NeuroEngineering and Rehabilitation	2017	23 (12/11)	Subacute; C2–L4; AIS C–D	Overground robotic exoskeleton-assisted gait training (HANK)	Traditional overground gait training	LEMS; WISCI-II; 10MWT; TUG; 6MWT; SCIM-III
Hitzig (2013) [19]	Topics in Spinal Cord Injury Rehabilitation	N/A	34 (17/17)	Chronic; C2–T12; AIS C–D	FES-assisted walking + body-weight-supported treadmill training	Aerobic + resistance exercise programme	SCIM mobility sub-score; SWLS; Lawton IADL; CHART; RNL Index
Kapadia (2014) [23]	The Journal of Spinal Cord Medicine	N/A	34 (17/17)	Chronic; C2–T12; AIS C–D	FES-assisted walking + body-weight-supported treadmill training	Aerobic + resistance exercise programme	SCIM mobility sub-score; 10MWT; 2/4/6MWT; TUG; FIM locomotor score; MAS; pendulum test
Kryger (2019) [26]	Journal of Medical Internet Research	2011 (EHEALTH)	38 (19/19)	Chronic; cervical-thoracic	Interactive Mobile Health and Rehabilitation (iMHere) system + standard care	Standard care	Number of UTIs; Pressure injuries; ED visits; hospitalizations; COPM; AMIS-II; BDI-II; WHOQOL-BREF; CHART-SF
Piira (2020) [25]	Spinal Cord	2010	37 (16/21)	Chronic; cervical/thoracic/lumbar; AIS C–D	Body weight supported locomotor training (with treadmill or Lokomat® gait training robot)	Usual care	Health-related quality of life (SF-36 PCS/MCS)*; LEMS; 10MWT; 6MWT; BBS; IPAQ-SF; EBSE; BREQ
Spungen (2024) [22]	JAMA Network Open	2010	161 (78/83)	Chronic; cervical-thoracic	Home- and community-based exoskeletal-assisted walking (ReWalk®)	Standard of care wheelchair use	MCS/VR-36*; SCI-QOL*; Body fat mass; PROMIS sleep disturbance; SCI-FI; SCI-QOL emotional health; social participation; metabolic outcomes
Xiang (2023) [24]	IEEE Transactions on Neural Systems and Rehabilitation Engineering	N/A	40 (20/20)	Chronic; T4–L1; AIS A–C	Exoskeleton-assisted walking (AIDER powered exoskeleton)	Conventional exercise programme	Pulmonary ventilation function (FVC, predicted FVC%, FEV1)*; TCT; LEMS; MAS; cartilage thickness; bone mineral density; MBI; 6MWT; 10MWT; adverse events

AIS American spinal injury association impairment scale, AMIS-II Adolescent self-management and independence scale-II, BBS berg balance scale, BDI-II beck depression inventory-II, BREQ behavioural regulation in exercise questionnaire, CHART craig handicap assessment and reporting technique, CHART-SF craig handicap assessment and reporting technique short form, COPM Canadian occupational performance measure, EBSE exercise barriers self-efficacy, ED emergency department, FES functional electrical stimulation, FEV1 forced expiratory volume in 1 s, FIM functional independence measure, FVC forced vital capacity, IPAQ-SF international physical activity questionnaire short form, LEMS lower extremity motor score, MAS modified ashworth scale, MBI modified barthel index, MCS mental component summary, MEPs motor evoked potentials, PCS physical component summary, PROMIS patient-reported outcomes measurement information system, RNL Index reintegration to normal living index, SCIM-III spinal cord independence measure III, SCI-FI spinal cord injury functional index, SCI-QOL spinal cord injury quality of life, SF-36 36-Item short form health survey, SWLS satisfaction with life scale, tSCS transcutaneous spinal cord stimulation, TCT trunk control test, TUG timed up and go, UTIs urinary tract infections, WISCI-II walking index for spinal cord injury II, 10MWT 10 meter walk test, 6MWT 6 min walk test, VR-36 veterans RAND 36-item health survey, WHOQOL-BREF world health organization quality of life brief instrument.

Regarding TBR interventions, three studies [21, 24, 25] investigated robotic or exoskeleton-assisted gait training, two studies [19, 23] evaluated FES-assisted walking, one study [20] combined transcutaneous spinal cord stimulation with robotic-assisted gait training, one study [26] investigated an interactive mobile health rehabilitation system, and one study [22] examined home- and community-based exoskeletal-assisted walking.

Comparator interventions were non-technology-based in seven studies [19, 21–26], whereas one study used sham transcutaneous spinal cord stimulation combined with robotic-assisted gait training [20].

The included studies primarily reported outcomes related to motor function and/or mobility. Six studies [19–21, 23, 24, 26] also included measures of functional independence, self-management, or occupational performance, whereas three studies [22, 25, 26] assessed health-related quality of life. Four trials explicitly declared a primary outcome, which included measures of motor impairment [20], health-related quality of life [22, 25], and pulmonary outcomes [24]. In the remaining four studies [19, 21, 23, 26], no clear distinction between primary and secondary outcomes was reported.

Five studies specified the CONSORT version used, including three trials [20, 22, 25] referring to CONSORT 2010 [27], one trial [21] referring to the CONSORT 2017 non-pharmacological extension [28], and one trial [26] referring to the CONSORT-EHEALTH 2011 extension [29]. The remaining three studies [19, 23, 24] cited the CONSORT statement without specifying a version or year.

### CHAMP evaluation

**Fig. 2:**
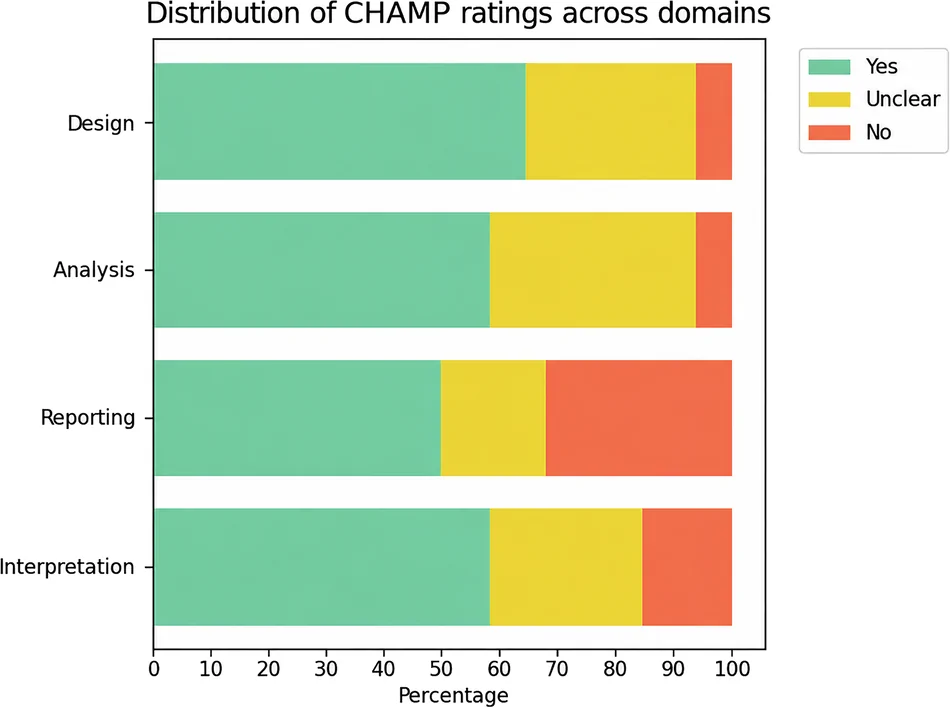
Distribution of CHAMP item ratings across domains. Percentages are calculated based on all assessed items within each domain across the included studies.

CHAMP performance varied across domains (Fig. 2) and individual items (Table 2; study-level assessments are reported in Supplement S2). Compliance was highest in the design domain (64.6%), followed by the interpretation (58.9%) and analysis (58.8%) domains, whereas the reporting domain showed the lowest proportion of fulfilled items (50.0%) and the highest proportion of not fulfilled ratings.

**Table 2:** Item-level assessment of statistical reporting quality using the CHAMP checklist across included studies.

Domain	Item	Description	Yes	No	Unclear
Design					
	1	Clear description of the goal of research, study objective(s), study design and study population	7 (87.5%)	0 (0.0%)	1 (12.5%)
	2	Clear description of outcomes, exposures/treatments and covariates, and their measurement methods	8 (100%)	0 (0.0%)	0 (0.0%)
	3	Validity of the study design	7 (87.5%)	0 (0.0%)	1 (12.5%)
	4	Clear statement and justification of sample size	4 (50.0%)	1 (12.5%)	3 (37.5%)
	**5**	Clear declaration of design violations and acceptability of the design violations	3 (37.5%)	1 (12.5%)	4 (50.0%)
	**6**	Consistency between the paper and its previously published protocol	2 (25.0%)	1 (12.5%)	5 (62.5%)
Analysis					
	7	Correct and complete description of statistical methods	8 (100%)	0 (0.0%)	0 (0.0%)
	8	Valid statistical methods used and assumptions outlined	5 (62.5%)	0 (0.0%)	3 (37.5%)
	9	Appropriate assessment of treatment effect or interaction between treatment and another covariate	7 (87.5%)	1 (12.5%)	0 (0.0%)
	10	Correct use of correlation and associational statistical testing	7 (87.5%)	0 (0.0%)	1 (12.5%)
	11	Appropriate handling of continuous predictors	6 (75.0%)	0 (0.0%)	2 (25.0%)
	**12**	CIs do not include impossible values	1 (12.5%)	0 (0.0%)	7 (87.5%)
	**13**	Appropriate comparison of baseline characteristics between the study arms in randomised trials	2 (25.0%)	4 (50.0%)	2 (25.0%)
	**14**	Correct assessment and adjustment of confounding	1 (12.5%)	0 (0.0%)	7 (87.5%)
	15	Avoiding model extrapolation not supported by data	7 (87.5%)	0 (0.0%)	1 (12.5%)
	**16**	Adequate handling of missing data	3 (37.5%)	0 (0.0%)	5 (62.5%)
Reporting					
	17	Adequate and correct description of the data	8 (100%)	0 (0.0%)	0 (0.0%)
	**18**	Descriptive results provided as occurrence measures with CIs and analytical results provided as association measures and CIs along with *p* values	0 (0.0%)	6 (75.0%)	2 (25.0%)
	**19**	CIs provided for the contrast between groups rather than for each group	1 (12.5%)	7 (87.5%)	0 (0.0%)
	20	Avoiding selective reporting of analyses and p-hacking	5 (62.5%)	1 (12.5%)	2 (25.0%)
	**21**	Appropriate and consistent numerical precision for effect sizes, test statistics and p values, and reporting the p values rather than their range	3 (37.5%)	4 (50.0%)	1 (12.5%)
	22	Providing sufficient numerical results that could be included in a subsequent meta-analysis	7 (87.5%)	0 (0.0%)	1 (12.5%)
	23	Acceptable presentation of figures and tables	4 (50.0%)	0 (0.0%)	4 (50.0%)
Interpretation					
	24	Interpreting the results based on association measures and 95% CIs along with p values, and correctly interpreting large p values as indecisive results	4 (50.0%)	2 (25.0%)	2 (25.0%)
	**25**	Using CIs rather than post hoc power analysis for interpreting the results of studies	1 (12.5%)	7 (87.5%)	0 (0.0%)
	26	Correctly interpreting occurrence or association measures	5 (62.5%)	0 (0.0%)	3 (37.5%)
	27	Distinguishing causation from association and correlation	6 (75.0%)	0 (0.0%)	2 (25.0%)
	28	Results of prespecified analyses are distinguished from the results of exploratory analyses in the interpretation	4 (50.0%)	0 (0.0%)	4 (50.0%)
	29	Appropriate discussion of the study methodological limitations	8 (100%)	0 (0.0%)	0 (0.0%)
	30	Drawing only conclusions supported by the statistical analysis and no generalisation of the results to subjects outside the target population	5 (62.5%)	0 (0.0%)	3 (37.5%)

Values are presented as number (percentage). Each item was rated as fulfilled (Yes), not fulfilled (No), or unclear. Items are grouped by CHAMP domains (design, analysis, reporting, and interpretation). Items highlighted in bold indicate critical methodological or reporting issues, defined as those with low compliance (<50% fulfilled).

Full compliance (100%) was observed for several items across domains, including the clear description of outcomes and measurement methods (item 2), statistical methods (item 7), data reporting (item 17), and discussion of methodological limitations (item 29), indicating that these aspects were consistently well reported across all included studies.

Within the analysis domain, despite the relatively high overall compliance, variability across individual items was observed. The plausibility of confidence intervals (item 12) was fulfilled in 12.5% of studies [20] and rated as unclear in 87.5% [19, 21–26]. Assessment of baseline comparability (item 13) was fulfilled in 25.0% of studies [20, 24], not fulfilled in 50.0% [19, 21, 23, 26], and rated as unclear in 25.0% [22, 25]. Adjustment for confounding (item 14) was fulfilled in 12.5% of studies [20] and rated as unclear in 87.5% [19, 21–26]. Handling of missing data (item 16) was fulfilled in 37.5% of studies [20, 22, 24] and rated as unclear in 62.5% [19, 21, 23, 25, 26].

In the design domain, sample size justification (item 4) was reported in 50.0% of studies [21, 22, 24, 26], not fulfilled in 12.5% [20], and rated as unclear in 37.5% [19, 23, 25]. Reporting of protocol adherence and design deviations showed lower levels of clarity: item 5 was fulfilled in 37.5% of studies [19, 22, 24], not fulfilled in 12.5% [20], and rated as unclear in 50.0% [21, 23, 25, 26], while item 6 was fulfilled in 25.0% of studies [22, 24], not fulfilled in 12.5% [21], and rated as unclear in 62.5% [19, 23, 25, 26].

The reporting domain showed the lowest proportion of fulfilled items. Between-group confidence intervals (item 19) were reported in 12.5% of studies [20] and not fulfilled in 87.5% [19, 21–26]. Complete reporting of results including both confidence intervals and *p*-values (item 18) was not observed in any study, with 75.0% of studies rated as not fulfilled [19, 21–23, 25, 26] and 25.0% as unclear [20, 24]. Precision of numerical reporting (item 21) was fulfilled in 37.5% of studies [19, 21, 24], not fulfilled in 50.0% [20, 22, 25, 26], and rated as unclear in 12.5% [23].

In the interpretation domain, correct use of confidence intervals (item 25) was fulfilled in 12.5% of studies [20] and not fulfilled in 87.5% [19, 21–26], representing one of the most critical items in this domain. The distinction between pre-specified and exploratory analyses (item 28) was fulfilled in 50.0% of studies [21, 22, 24, 26] and rated as unclear in the remaining 50.0% [19, 20, 23, 25].

Inter-rater agreement was 74.2% (178/240 ratings), with an overall unweighted Cohen’s κ of 0.14 (95% CI [0.01, 0.26]). Domain-specific percentage agreement ranged from 71.4–81.2%, whereas Cohen’s κ coefficients ranged from 0.00–0.37. The highest agreement was observed for the design domain (81.2%; κ = 0.37, 95% CI [0.08, 0.66]), followed by the reporting (71.4%; κ = 0.19, 95% CI [−0.07, 0.45]), analysis (72.5%; κ = 0.06, 95% CI [−0.14, 0.26]), and interpretation (73.2%; κ = 0.00, 95% CI [0.00, 0.00]) domains. Detailed inter-rater agreement results for each CHAMP domain are presented in Supplement S3.

### Risk of bias assessment

**Fig. 3:**
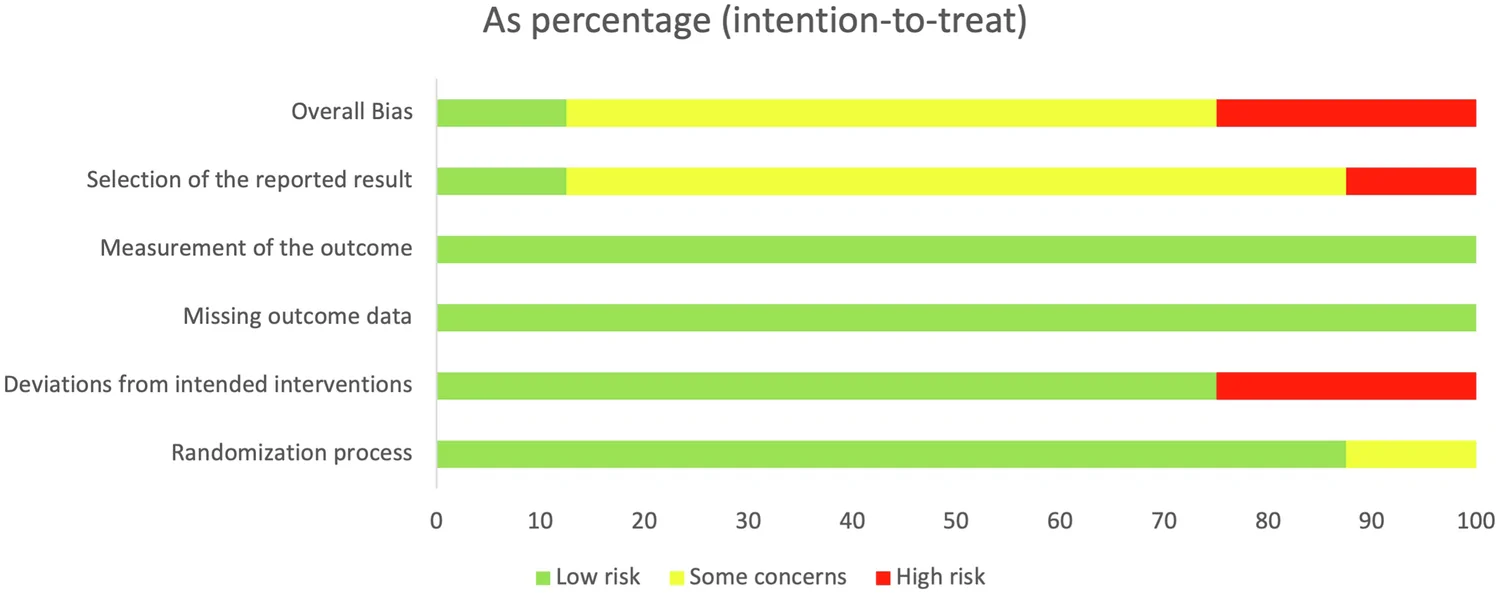
Distribution of RoB 2 judgements across domains and overall assessment, expressed as percentages.

At the domain level, all studies were judged at low risk of bias for missing outcome data (D3) and measurement of the outcome (D4), indicating adequate handling of attrition and outcome assessment. For the randomisation process (D1), seven studies [19–25] were rated at low risk of bias, while one study [26] raised some concerns due to insufficient reporting of allocation concealment. Higher risk judgements were observed in other domains. Deviations from intended interventions (D2) were rated at high risk in two studies [19, 23] because the delivered intervention deviated from the intended intervention. Selection of the reported result (D5) was rated at high risk in one study [23] due to inconsistencies between the published protocol and the final report regarding sample size and assessed outcomes. In addition, six studies [19–21, 24–26] raised some concerns in D5 because no detailed statistical analysis plan was available in the published protocol, while only one study [22] was judged at low risk of bias for this domain. The RoB 2 assessment is summarised in Fig. 3. Overall, one study [22] was judged at low risk of bias (12.5%), five studies [20, 21, 24–26] raised some concerns (62.5%), and two studies [19, 23] were judged at high risk of bias (25.0%).

### Comparison of CHAMP performance across RoB 2 judgements

Further descriptive comparisons were conducted to examine how CHAMP scores varied across studies grouped by overall RoB 2 judgement. Among studies classified as having some concerns, CHAMP scores ranged widely, from 0.30 [25] to 0.83 [24] (Table 3), indicating substantial variability in statistical reporting quality despite similar RoB 2 classifications. Studies judged at high risk of bias also showed heterogeneous CHAMP scores (0.30 and 0.67). Given this variability and the absence of formal statistical testing, no clear alignment between CHAMP performance and RoB 2 classification could be identified.

**Table 3:** CHAMP performance and overall risk of bias (RoB 2) classification across included studies.

Study	CHAMP	RoB 2
Xiang (2023) [24]	0.83	Some concerns
Comino-Suárez (2025) [20]	0.77	Some concerns
Spungen (2024) [22]	0.70	Some concerns
Hitzig (2013) [19]	0.67	High
Gil-Agudo (2023) [21]	0.63	Some concerns
Kryger (2019) [26]	0.43	Some concerns
Kapadia (2014) [23]	0.30	High
Piira (2020) [25]	0.30	Some concerns

CHAMP scores are expressed as the proportion of fulfilled items (number of items rated as “Yes” divided by the total number of applicable items).

## Discussion

This meta-research study provides a structured evaluation of statistical quality in TBR trials for adults with SCI. Overall, substantial variability in CHAMP performance was observed, with scores ranging from 0.30–0.83 (1 indicating all items fulfilled). This variability was evident across domains, with lower levels of compliance particularly observed in reporting and interpretation.

No clear alignment was observed between statistical quality (CHAMP) and overall risk of bias (RoB 2), based on descriptive comparisons. Given the limited number of studies, these analyses should be interpreted as exploratory only. Studies classified as having some concerns showed substantial variability in CHAMP scores, ranging from 0.30–0.83, suggesting that RoB 2 and CHAMP may capture complementary but distinct aspects of trial quality. While the RoB 2 tool primarily evaluates risks affecting internal validity [11], CHAMP provides a more detailed appraisal of the transparency and completeness of statistical design, analysis, reporting, and interpretation [13]. Consequently, RoB 2 may identify broader concerns related to reporting and protocol consistency, whereas CHAMP further characterises the specific statistical limitations underlying these concerns.

An additional consideration concerns the conceptual differences between CHAMP and RoB 2 tools. RoB 2 relies on a structured algorithm in which specific domains can disproportionately influence the overall judgement, whereas CHAMP is a checklist assessing statistical transparency without predefined weighting of items. In this study, CHAMP items were summarised into a single score assigning equal weight to each item. While this approach facilitates comparison across studies, it does not account for the relative importance of individual statistical aspects. These differences should be considered when interpreting the lack of alignment between CHAMP performance and RoB 2 classifications.

From a practical perspective, incomplete or unclear statistical reporting may limit the interpretability and usability of trial findings. Several critical issues emerged at the item level, including the frequent absence of between-group confidence intervals, incomplete reporting of *p*-values, and insufficient distinction between pre-specified and exploratory analyses. These limitations may hinder a proper understanding of the magnitude, precision, and robustness of treatment effects, ultimately affecting reproducibility and the integration of findings into evidence syntheses.

The frequent lack of clarity regarding consistency between study reports and their protocols, together with the absence of detailed statistical analysis plans in several published protocols, may indicate that limitations in statistical transparency originate early in the research process rather than emerging solely at the reporting stage. In this context, the concerns identified by RoB 2 in the domain related to selection of the reported result (D5) appear to reflect broader issues that are further detailed through the CHAMP assessment.

Notably, a considerable number of potentially eligible RCTs were excluded during the screening process because they did not explicitly report adherence to the CONSORT statement. Although the absence of an explicit declaration does not necessarily indicate poor reporting quality, it may reflect suboptimal reporting practices or limited transparency regarding adherence to established reporting standards. This observation further reinforces the need to promote greater transparency and more consistent reporting practices in SCI rehabilitation research.

These results align with previous meta-research showing suboptimal adherence to reporting standards in clinical and rehabilitation research [7], and extend this evidence by specifically focusing on statistical aspects through a structured, item-level approach. Improving statistical transparency should therefore be considered a key step to enhance the interpretability and reproducibility of rehabilitation trials.

These findings can be interpreted in light of current recommendations emphasising the importance of reporting effect sizes, confidence intervals, and measures of precision to support clinical interpretation [30]. In this context, our results indicate a misalignment with these recommendations, as key elements such as between-group confidence intervals and complete reporting of results were frequently absent. Given that confidence intervals are essential for evaluating the magnitude, precision, and uncertainty surrounding treatment effects, their omission may limit the interpretability, reliability, and clinical applicability of findings. These limitations may also affect the reproducibility of the reported analyses and, in some cases, the robustness of effect estimation. Taken together, such issues may contribute not only to delays in translating research findings into clinical practice, but also to difficulties in synthesising evidence and formulating strong clinical recommendations. Inconsistent statistical reporting and limited transparency may reduce study reproducibility and comparability across trials, thereby increasing heterogeneity in evidence synthesis and weakening confidence in the overall body of evidence.

### Limitations

This study has several limitations that must be acknowledged. First, the small number of included studies limited the possibility of conducting formal statistical analyses and restricted the generalisability of the findings. This was partly driven by the strict inclusion criteria, which were restricted to RCTs explicitly declaring adherence to the CONSORT statement. While this approach aimed to focus on studies expected to follow recognised reporting standards, it may have reduced the pool of eligible studies and excluded trials with adequate statistical reporting but without an explicit CONSORT adherence declaration.

Second, the CHAMP scoring approach involved the transformation of categorical ratings into a numerical summary score, a procedure that was developed ad hoc for this study and has not been formally validated. Although this approach allowed a quantitative synthesis of statistical reporting quality, it may have introduced some simplification of the underlying information.

These limitations suggest that the findings should be interpreted as exploratory and hypothesis-generating rather than confirmatory.

## Conclusion

Statistical quality in TBR trials for SCI shows substantial variability. Although RoB 2 and CHAMP assess different dimensions of study quality, concerns identified in the RoB 2 domain related to selection of the reported result were frequently accompanied by limitations in statistical transparency identified through CHAMP. These findings suggest that weaknesses in statistical reporting may originate early in the research process, including at the protocol and analysis-planning stage.

These findings highlight the need for greater attention to the quality of statistical design, analysis, and reporting in rehabilitation research. Integrating CONSORT with structured tools such as CHAMP may support both reviewers and researchers in achieving a more comprehensive evaluation of research quality, enhancing the interpretability and reproducibility of rehabilitation research, and guiding the critical appraisal as well as the planning and reporting of future studies.

## Supplementary information

Supplement

## Data Availability

All data analysed during this study are included in this article and its supplementary information files.
