# Supplementary material for: Statistical quality in RCTs of technology-based rehabilitation after spinal cord injury: A CHAMP-Based Meta-Research Study
Source: Spinal Cord. 2026 Sep 18;64(10):874–82. doi: 10.1038/s41393-026-01276-0 (PMC13668654; doi:10.1038/s41393-026-01276-0)
Supplement: Supplementary file 1 [file 41393_2026_1276_MOESM1_ESM.pdf]

This supplementary file includes the complete search strategy (S1), the study-level CHAMP assessments across all checklist items (S2), and the inter-rater agreement results for the CHAMP checklist assessment (S3).

S1 – Complete search strategy

MEDLINE

#1

"Spinal Cord Injuries"[MeSH Terms] OR "spinal cord injury"[Title/Abstract] OR "Spinal Cord Injuries"[Title/Abstract] OR "spinal cord lesion"[Title/Abstract] OR "spinal cord trauma"[Title/Abstract] OR "traumatic spinal cord injury"[Title/Abstract] OR "SCI"[Title/Abstract]

AND #2

("rehabilitation"[MeSH Terms] OR "rehabilitation"[Title/Abstract] OR "physical therapy"[Title/Abstract] OR "physiotherapy"[Title/Abstract]) AND ("robotics"[MeSH Terms] OR "robotic"[Title/Abstract] OR "robot-assisted"[Title/Abstract] OR "exoskeleton"[Title/Abstract] OR "end effector"[Title/Abstract] OR "virtual reality"[MeSH Terms] OR "VR"[Title/Abstract] OR "augmented reality"[Title/Abstract] OR "AR"[Title/Abstract] OR "brain-computer interfaces"[MeSH Terms] OR "brain-computer interface"[Title/Abstract] OR "BCI"[Title/Abstract] OR "EMG-driven"[Title/Abstract] OR "human-machine interface"[Title/Abstract] OR "biofeedback"[Title/Abstract] OR "telerehabilitation"[MeSH Terms] OR "telerehabilitation"[Title/Abstract] OR "tele-rehabilitation"[Title/Abstract] OR "remote rehabilitation"[Title/Abstract] OR "mobile application"[Title/Abstract] OR "wearable device"[Title/Abstract] OR "sensor-based"[Title/Abstract] OR "interactive platform"[Title/Abstract] OR "video-based exercise"[Title/Abstract])

AND #3

"randomized controlled trial"[Publication Type] OR "controlled clinical trial"[Publication Type] OR "Randomized"[Title/Abstract] OR "Placebo"[Title/Abstract] OR "drug therapy"[MeSH Subheading] OR "Randomly"[Title/Abstract] OR "Trial"[Title/Abstract] OR "Groups"[Title/Abstract]

CINAHL Complete

(MH "Spinal Cord Injuries" OR XB "spinal cord injury" OR XB "spinal cord injuries" OR XB "spinal cord lesion" OR XB "spinal cord trauma" OR XB "traumatic spinal cord injury" OR XB "SCI") TX

AND

(rehabilitation OR "physical therapy" OR physiotherapy) TX

AND
(robotics OR "robot-assisted" OR "robot-assisted therapy" OR exoskeleton OR end-effector OR "virtual reality" OR "augmented reality" OR "brain computer interface" OR "human-machine interface" OR "EMG-driven" OR telerehabilitation OR tele-rehabilitation OR "remote rehabilitation" OR biofeedback OR "mobile application" OR "wearable device" OR sensor-based OR "interactive platform" OR "video-based exercise") TX
AND
("Randomized Controlled Trial" OR "Controlled Clinical Trial" OR "Randomized" OR "Placebo" OR "Drug Therapy" OR "Randomly" OR "Trial" OR "Groups") Publication Type
Limiters: Published Date from 28/08/1996 to present, Human

Cochrane CENTRAL

ID Search Hits
#1 (spinal cord injury):ti,ab,kw
#2 (spinal cord injuries):ti,ab,kw
#3 (spinal cord lesion):ti,ab,kw
#4 (spinal cord trauma):ti,ab,kw
#5 (traumatic spinal cord injury):ti,ab,kw
#6 (SCI):ti,ab,kw
#7 MeSH descriptor: [Rehabilitation] explode all trees
#8 (rehabilitation):ti,ab,kw
#9 MeSH descriptor: [Physical Therapy Modalities] explode all trees
#10 ("physical therapy"):ti,ab,kw
#11 (physiotherap*):ti,ab,kw
#12 #1 OR #2 OR #3 OR #4 OR #5 OR #6
#13 #7 OR #8 OR #9 OR #10 OR #11
#14 MeSH descriptor: [Robotics] explode all trees
#15 (robotic*):ti,ab,kw
#16 (robot-assisted NEXT therapy):ti,ab,kw
#17 (robot-assisted):ti,ab,kw
#18 MeSH descriptor: [Exoskeleton Device] explode all trees
#19 (exoskeleton*):ti,ab,kw
#20 (end-effector*):ti,ab,kw
#21 (virtual NEXT reality):ti,ab,kw

#22 MeSH descriptor: [Virtual Reality] explode all trees

#23 (VR):ti,ab,kw

#24 MeSH descriptor: [Augmented Reality] explode all trees

#25 (augmented NEXT reality):ti,ab,kw

#26 (AR):ti,ab,kw

#27 MeSH descriptor: [Brain-Computer Interfaces] explode all trees

#28 (Brain NEXT computer NEXT Interface*):ti,ab,kw

#29 (BCI):ti,ab,kw

#30 (EMG-driven):ti,ab,kw

#31 ("human machine interface"):ti,ab,kw

#32 MeSH descriptor: [Biofeedback, Psychology] explode all trees

#33 ("biofeedback"):ti,ab,kw

#34 MeSH descriptor: [Telerehabilitation] explode all trees

#35 ("telerehabilitation"):ti,ab,kw

#36 ("tele-rehabilitation"):ti,ab,kw

#37 (remote NEXT rehabilitation):ti,ab,kw

#38 (mobile NEXT application*):ti,ab,kw

#39 MeSH descriptor: [Wearable Electronic Devices] explode all trees

#40 (wearable NEXT device*):ti,ab,kw

#41 (sensor-based):ti,ab,kw

#42 (interactive NEXT platform):ti,ab,kw

#43 (video-based NEXT exercise*):ti,ab,kw

#44 #14 OR #15 OR #16 OR #17 OR #18 OR #19 OR #20 OR #21 OR #22 OR #23 OR #24 OR #25 OR #26 OR #27 OR #28 OR #29 OR #30 OR #31 OR #32 OR #33 OR #34 OR #35 OR #36 OR #37 OR #38 OR #39 OR #40 OR #41 OR #42 OR #43

#45 ("randomized controlled trial"):ti,ab,kw

#46 ("controlled clinical trial"):ti,ab,kw

#47 (randomized):ti,ab,kw

#48 (placebo):ti,ab,kw

#49 MeSH descriptor: [Randomized Controlled Trial] explode all trees

#50 (randomly):ti,ab,kw

#51 (groups):ti,ab,kw

#52 #45 OR #46 OR #47 OR #48 OR #49 OR #50 OR #51

#53 #12 AND #13 AND #44 AND #52 with Cochrane Library publication date Between Aug 1996 and xxx

PEDro

Abstract & Title: spinal cord injury

Subdiscipline: neurology

Method: clinical trial

New records added since: 28/08/1996

When searching: Match all search terms (AND)

Web of Science

#1

TS=("Spinal Cord Injuries" OR "spinal cord injury" OR "spinal cord lesion" OR "spinal cord trauma" OR "traumatic spinal cord injury" OR "SCI")

AND #2

TS=("rehabilitation" OR "physical therap*" OR "physiotherap*")

AND #3

TS=("robotics" OR "robot-assisted therap*" OR "exoskeleton*" OR "end-effector*" OR "virtual reality" OR "augmented reality" OR "brain computer interface*" OR "EMG-driven" OR "human-machine interface*" OR "biofeedback" OR "telerehabilitation" OR "tele-rehabilitation" OR "remote rehabilitation" OR "mobile application*" OR "wearable device*" OR "sensor-based" OR "interactive platform*" OR "video-based exercise*")

AND #4

TS=(Trial*)

S2 – Study-level CHAMP assessments across all checklist items

ID	Comino-Suárez 2025	Gil-Agudo 2023	Hitzig 2013	Kapadia 2014	Kryger 2019	Piira 2020	Spungen 2024	Xiang 2023
**1**	Yes	Yes	Yes	Yes	Yes	Unclear	Yes	Yes
**2**	Yes	Yes	Yes	Yes	Yes	Yes	Yes	Yes
**3**	Yes	Yes	Yes	Yes	Yes	Unclear	Yes	Yes
**4**	No	Yes	Unclear	Unclear	Yes	Unclear	Yes	Yes
**5**	No	Unclear	Yes	Unclear	Unclear	Unclear	Yes	Yes
**6**	Unclear	No	Unclear	Unclear	Unclear	Unclear	Yes	Yes
**7**	Yes	Yes	Yes	Yes	Yes	Yes	Yes	Yes
**8**	Yes	Yes	Yes	Unclear	Unclear	Unclear	Yes	Yes
**9**	Yes	Yes	Yes	No	Yes	Yes	Yes	Yes
**10**	Yes	Yes	Yes	Unclear	Yes	Yes	Yes	Yes
**11**	Unclear	Unclear	Yes	Yes	Yes	Yes	Yes	Yes
**12**	Yes	Unclear	Unclear	Unclear	Unclear	Unclear	Unclear	Unclear
**13**	Yes	No	No	No	No	Unclear	Unclear	Yes
**14**	Yes	Unclear	Unclear	Unclear	Unclear	Unclear	Unclear	Unclear
**15**	Yes	Yes	Yes	Yes	Yes	Unclear	Yes	Yes
**16**	Yes	Unclear	Unclear	Unclear	Unclear	Unclear	Yes	Yes
**17**	Yes	Yes	Yes	Yes	Yes	Yes	Yes	Yes
**18**	Unclear	No	No	No	No	No	No	Unclear
**19**	Yes	No	No	No	No	No	No	No
**20**	Yes	Yes	Yes	Unclear	No	Unclear	Yes	Yes
**21**	No	Yes	Yes	Unclear	No	No	No	Yes
**22**	Yes	Yes	Yes	Yes	Yes	Yes	Unclear	Yes
**23**	Yes	Yes	Yes	Unclear	Unclear	Unclear	Unclear	Yes
**24**	Yes	No	Yes	Unclear	Unclear	No	Yes	Yes
**25**	Yes	No	No	No	No	No	No	No
**26**	Yes	Yes	Yes	Unclear	Unclear	Unclear	Yes	Yes
**27**	Yes	Yes	Yes	Unclear	Unclear	Yes	Yes	Yes

**28**	Unclear	Yes	Unclear	Unclear	Yes	Unclear	Yes	Yes
**29**	Yes	Yes	Yes	Yes	Yes	Yes	Yes	Yes
**30**	Yes	Yes	Yes	Unclear	Unclear	Unclear	Yes	Yes

S3 – Inter-rater agreement results for the CHAMP checklist assessment

Domain	Items	Agreement (%)	Cohen's κ	95% CI
**DESIGN**	1-6	81.2	0.37	[0.08; 0.66]
**ANALYSIS**	7-16	72.5	0.06	[-0.14; 0.26]
**REPORTING**	17-23	71.4	0.19	[-0.07; 0.45]
**INTERPRETATION**	24-30	73.2	0	[0.00; 0.00]

Percentage agreement and unweighted Cohen's κ coefficients between the two independent reviewers for each CHAMP domain. Agreement was assessed considering the three possible ratings for each CHAMP item (Yes, No, and Unclear).
